# A species diversity dataset of beetles by three passive acquisition methods in Tei Tong Tsai (Hong Kong)

**DOI:** 10.1038/s41597-022-01310-9

**Published:** 2022-05-16

**Authors:** Shuzhe Zhao, Yijie Tong, Bei Teng, Xin Chen, Xingke Yang, Jing Li, Ming Bai

**Affiliations:** 1 College of Plant Protection, Hebei Agricultural University, Baoding, 071001 China; 2 Key Laboratory of Zoological Systematics and Evolution, Institute of Zoology, Chinese Academy of Sciences, Box 92, Beichen West Road, Chaoyang District, Beijing, 100101 China; 3 University of Chinese Academy of Sciences, Beijing, 100049 China; 4 Hainan Yazhou Bay Seed Lab, Building 1, No.7 Yiju Road, Yazhou District, Sanya City, Hainan Province, Sanya, 572024 China; 5 Cangzhou Normal University, Cangzhou, 061001 China; 6 Guangdong Key Laboratory of Animal Conservation and Resource Utilization, Guangdong Public Laboratory of Wild Animal Conservation and Utilization, Institute of Zoology, Guangdong Academy of Sciences, Guangzhou, 510260 China; 7 School of Agriculture, Ningxia University, Yinchuan, 750021 China

**Keywords:** Biodiversity, Ecosystem ecology

## Abstract

We based the dataset in this paper on the beetle collection from the sample site of Tei Tong Tsai (Hong Kong) from 1^st^ May to 28^th^ May 2019, a period of high insect diversity. A total of 16,270 beetles (photographed in 318 images) from 478 species belonging to 39 families were collected. The dataset consists of the following components: The original photo of the whole sample obtained at each site with each collection method, the morphological species identification chart, a statistical table describing the species and numbers of beetles collected on different dates at different sites using three passive acquisition methods, and a statistical table describing the longitude, latitude, and altitude information of each sampling point. We aimed to provide a database for the evaluation of beetle species diversity in Hong Kong and a paradigm for the effectiveness of passive acquisition in the beetle collection through the three representative methods, thus laying a foundation for biodiversity research.

## Abstract (summary)

**Table:** 

Measurement(s)	Geographic information and weather • Species identification • Beetles display • The number of beetles
Technology Type(s)	GPS and weather station (www.Nmc.cn) • Olympus EM5-II camera (with Micro Lens 60 mm F2.8) • Statistics module (Microsoft Excel 2016)
Sample Characteristic - Organism	Coleoptera
Sample Characteristic - Environment	Subtropical climate
Sample Characteristic - Location	Tei Tong Tsai (Hong Kong)

## Background & Summary

Biodiversity sustains human survival in many ways, especially in three major aspects: health, food, and industry. Therefore, the study of biodiversity has been a strong focus of ecological research and explored by scholars for a long time^1–3^. Many ecological studies on animal diversity have focused on mammals, birds, fish, and some insects^2,4–6^. As the largest group of insects, beetles are widespread and numerous. The evolution of beetles over billions of years has produced a wide variety of taxa. They occupy all ecological positions in space^7^, establishing beetles as a representative biodiversity research group and a longstanding subject for many scholars^7–9^.

Insect collection methods are an important part of our study, including active and passive methods. Due to their simple operation and easy portability, active collection methods are widely used, including net sweeping, striking, and vibration methods^7,10^. In recent years, passive acquisition methods such as pitfall, malaise, and light trapping have attracted the attention of many researchers for their high repeatability and large acquisition volume^11^. Beetles have diverse habitats, wide distribution, and complex life histories; as a result, they have diverse individual distribution spaces, which cause active collection methods to be less efficient in obtaining samples^10^. In addition, obtaining all taxa in a complex habitat with only one collection method is difficult. Using a combination of different collecting methods leads to more comprehensive and objective results^12^.

In 1989, Hallmann reported a 27-year study on insect diversity in 63 protected areas by trapping. The results showed a 75% decline in insect diversity over the 27 years. Among the reasons for this, habitat degradation and human disturbance are important factors that have caused widespread concern^13^. In this context, Ming Bai (Institute of Zoology, Chinese Academy of Sciences) and Alfried Vogler (Natural History Museum, London) initiated SITE100 to explore global insect diversity patterns in 100 large sample sites worldwide from three dimensions: species, morphological, and genetic diversity. Hong Kong (114°15′ E, 22°15′ N) is located along the coast of Southern China, with mountainous areas and country parks. Its suitable environment and geographic location have created a rich ecological environment in Hong Kong; therefore, it was listed as one of the sites in SITE100. The beautiful scenery and species richness designate Tei Tong Tsai in Hong Kong as a good experimental sample site for this study.

Thirteen sample sites were set up to evaluate local biodiversity and collect beetles in this study. Each sample site was equipped with a three-dimensional, passive acquisition method, including a flight interception trap (FIT), malaise trap (MT), and 10 pitfall traps (PT) covering the entire living space of the beetles. This arrangement ensured the objectivity of the evaluation results. Fixed sampling sites were selected in areas with low human disturbance to explore the biodiversity of the Tei Tong Tsai area. We intend to provide data support for local biodiversity studies through this dataset and assess the effects of the three passive acquisition methods presented by the SITE100 project.

## Methods

### Study sites

**Fig. 1:**
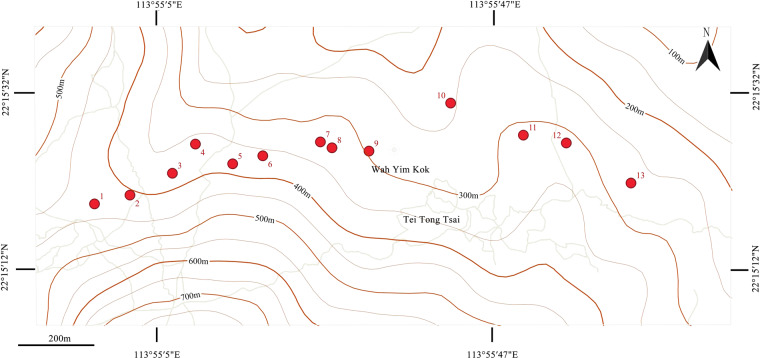
Sampling points for the three passive acquisition methods used in the Tei Tong Tsai sampling site (indicated by red dots).

The sample site Tei Tong Tsai is located within the Island District (112°5’ E, 22°5’ N Hong Kong, China) and connected to Lantau Country Park. The rich woods in Tei Tong Tsai provide a suitable environment for insects to survive, with rich biodiversity. Weather records (Supplement 1) for May 2019 show that the highefst temperature was 27.2 °C, the lowest was 15.7 °C, the average was 21.7 °C; and the annual average rainfall was 297.8 mm. The suitable temperature and rainfall have created a suitable ecological environment and high biodiversity, establishing Tei Tong Tsai as a prime location for studying beetle diversity. In May 2019, a 13 sample sites were selected for beetle collection (Fig. 1). All latitude and longitude formats were converted to degrees, minutes, and seconds.

### Experimental protocol

In this study, three passive collection methods were used for beetle collection. FIT is an efficient collecting method for insects with strong flying abilities and was first developed and used abroad^14^. MT and PT collect insects that are not strong flyers and live on the surface. A flight interception trap, a malaise trap, and 10 pitfall traps were set up to collect beetles in each sample site. Samples were selected to cover ecological environments at different longitudes, latitudes, altitudes, and distances from water sources. Reasonable sampling distances (depending on the terrain, with an interval between 100 and 200 m) were set up between sample sites to fully cover Tei Tong Tsai’s habitats. Due to the topography, the distance between the 10^th^ and 11^th^ sample points was about 350 m. The distance between two other close sample points were in the range of 100–200 m. All three traps were based on the original device to maximize the advantages and achieve better collection results.

**Fig. 2:**
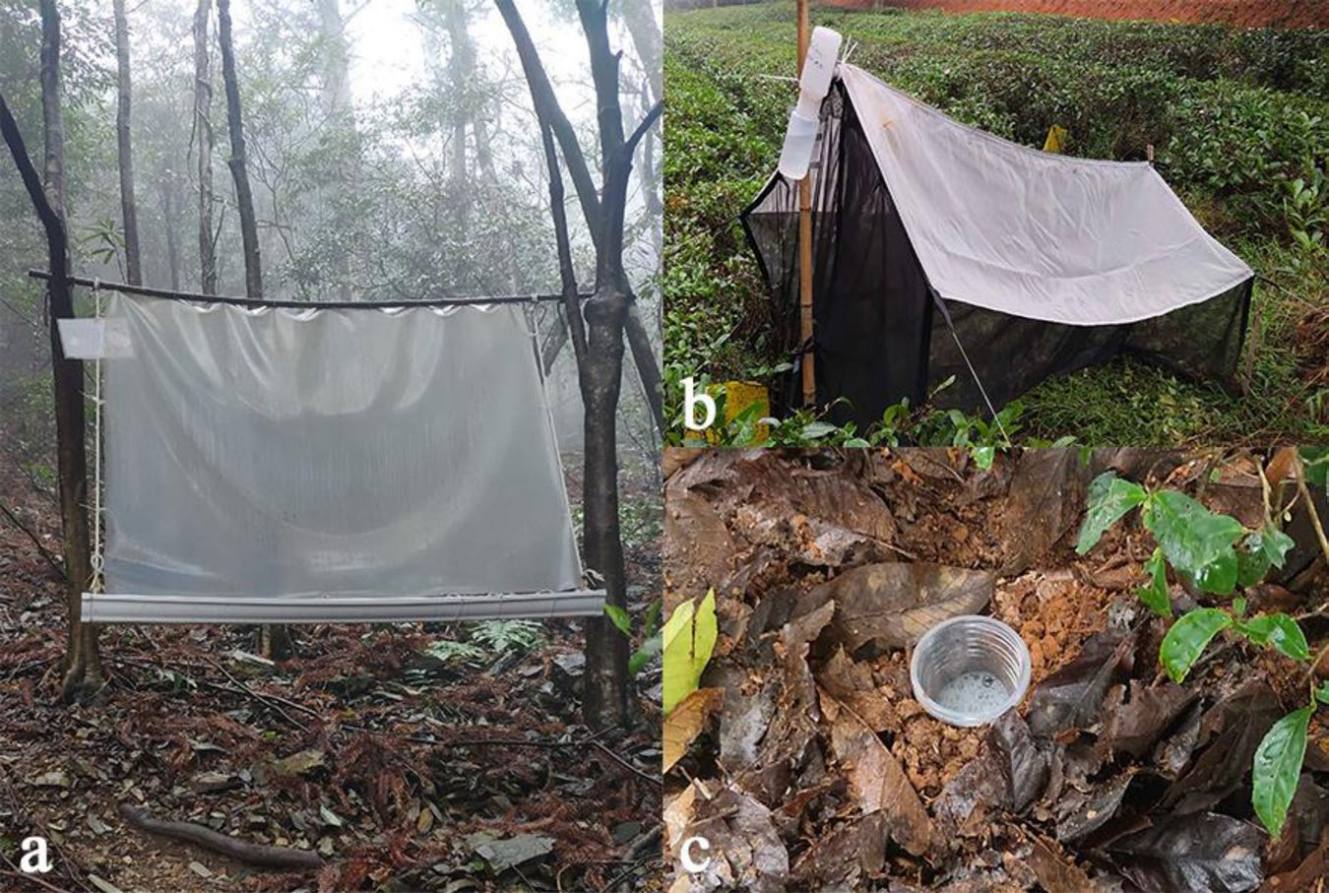
Three passive acquisition methods: (**a**) flight interception trap; (**b**) malaise trap; (**c**) pitfall trap.

Collection devices. The flight interception trap (Fig. 2a) mainly comprises an interceptor screen (plastic net, PVC plastic glass, or plexiglas) and an insect specimen receiver (PVC), which is an efficient collection device for intercepting and collecting insects with strong flight ability. The detailed installation steps include the following: Firstly, punch two holes on the long side of the PVC screen with a hole puncher spaced about 30 cm apart; then, fix the screen to a bamboo pole with silk, install the specimen receiver, fix all three, bolt the rope, and fix it in the air with a thick rope (the sink is about 0.5–1 m from the ground). After installation, relevant drugs were placed inside the specimen receiver to poison the insects. The drugs used depend on the purpose of the study. For morphological studies, saline (5 mmol/L NaCl solution) or water with detergent is used. By contrast, DNA molecular studies use a mixture of 2% SDS (sodium dodecyl sulfate) and EDTA (ethylene diamine tetraacetic acid, 0.1 mol/L, PH = 8) or highly concentrated alcohol, which effectively controls the degradation of DNA. Currently, high-concentration alcohol, SDS and EDTA mixtures are commonly used. The device is widely applicable and can be installed in almost any habitat; however, it is best installed along the insects’ flight paths, including roads, rivers, or creeks between valleys. In this experiment, we improved this device by increasing the size of the water trough considering the actual situation of the sample site. Also, to properly conduct the molecular experiments, the reagents we used were a mixture of SDS and EDTA. Therefore, the improved device was more suitable for diverse habitats, and the insect species collected were abundant, reflecting good collection practices^14^.

Malaise traps (Fig. 2b) are large tent-like structures constructed from thin mesh. They are among the most commonly used static non-attractant insect traps and insect collection devices. Invented by Malaise (1937) and later improved upon by Townes and Sharkey, these traps are important tools for insect collection and monitoring worldwide^15^. The malaise trap used at the Tei Tong Tsai Country Park was the Townes type, which is generally set up in forest areas with rich habitats and relatively stable ground. The material is usually meshed mosquito netting fabricated into a tent-shaped insect interception field. The insects hit the net vertically, continue to fly upward, and are gradually led into the trap by the tilted top. The drug in the trap is usually anhydrous ethanol, which intercepts beetles with weak flying abilities^16,17^.

The pitfall trap (Fig. 2c) is an effective method for capturing surface beetles; it is simple to use, easy to carry, and a common device for collection in the wild. The PT is created by digging a pit into the ground with the same depth as a wide-mouth plastic cup (20 cm high, 10 cm in diameter); The upper edge of the cup must be flushed with the soil surface, and a mixture of absolute ethanol is poured inside to collect flightless beetles^14^. About one-quarter of the way from the top, small holes are punched above the wide-mouth cup to prevent the loss of specimens from rainwater filling the cups. The 10 sets of traps in this experiment were not evenly distributed, but they were all in suitable habitats.

### Specimen sampling

The sampling site for this study was Tei Tong Tsai, and the sampling period was from 1^st^ May to 28^th^ May (2019). FIT, and PTs were collected once every two days. Due to the small number of beetles collected by MT, mt was collected only once. All beetles were picked out and arranged separately after collection, added to anhydrous ethanol, preserved, and labeled. The beetles collected by the three passive acquisition methods were picked according to morphological species.

#### Specimen identification

The taxonomic status for the family level of all samples was determined based on the relevant literature^18–21^. Relevant experts completed further identification (Supplement 2).

All the specimens collected in this study are currently in the zoological museum of the Institute of Zoology, Chinese Academy of Sciences (Beijing, China).

#### Specimen photography

**Fig. 3:**
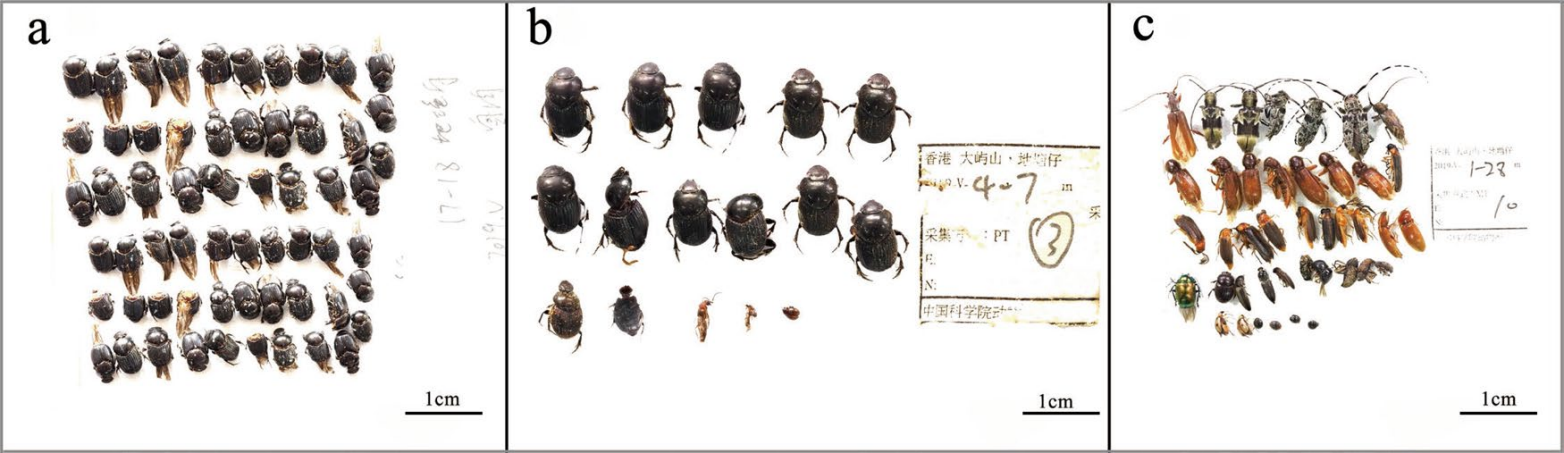
Examples of beetles collected from three passive acquisition methods: overall photos of beetles collected by (**a**) FIT, (**b**) PT, and (**c**) MT. On the bottom right corner shows scale in each photo.

Beetles were poured from the bottle and arranged separately according to the general species. Firstly, we used tweezers or a brush to place the beetles on unbreakable and unwrinkled paper (as far as possible with the backside upwards to keep them tight and neat, reducing the space left, and considering the label in the photograph). Simultaneously, we captured multiple photos according to the size and species of insect for the large specimens in the tube, adjusted the light near them to brighten the background, placed graph paper next to the beetles as a reference scale, then adjusted our Olympus camera settings to the appropriate photographing parameters. Finally, we inserted the photographed beetles and matching labels back into the tube and added anhydrous ethanol for preservation (Fig. 3). The labels were set in the photos as 2019 DTZ-FIT/MT/PTX-5XX-5XX (-N), in which 2019 represents the collection time, DTZ represents Tei Tong Tsai, FIT/MT/PT signifies the collection method, X represents the number of sampling points, 5XX-5XX represents sampling time, and N represents the photo number. If a sample site had many insects on the same date and required more than one photo, n was used to represent the number of photos. See the Supplement 3 for the complete document.

After the morphological data of the samples were collected, their Latin name and collection information were recorded in a table. Each passive acquisition method corresponded to a table, and each table was divided into 13 sheets according to 13 sampling points. The collection time was listed horizontally on each sheet, and the beetles’ species names were listed vertically (were named in the morphological species order as 1, 2, 3, …, N). The number of beetles was recorded in the corresponding position and the Supplement 4 file.

**Fig. 4:**
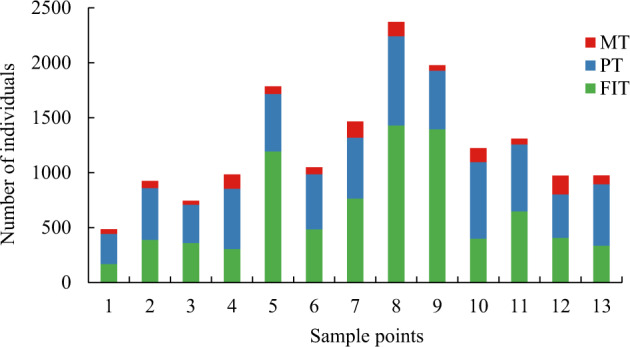
Data table of numbers of individual beetles collected by different methods at 13 sampling points. The red, blue, and green columns represent the number of beetles collected by MT, PT, and FIT, respectively.

**Fig. 5:**
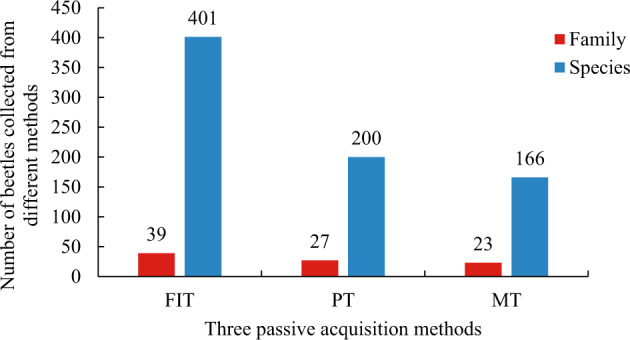
The number of beetles collected by different passive acquisition methods. Horizontal coordinates represent collection methods. The red column and blue column represent the number of beetles collected on the family level and species level, respectively.

**Fig. 6:**
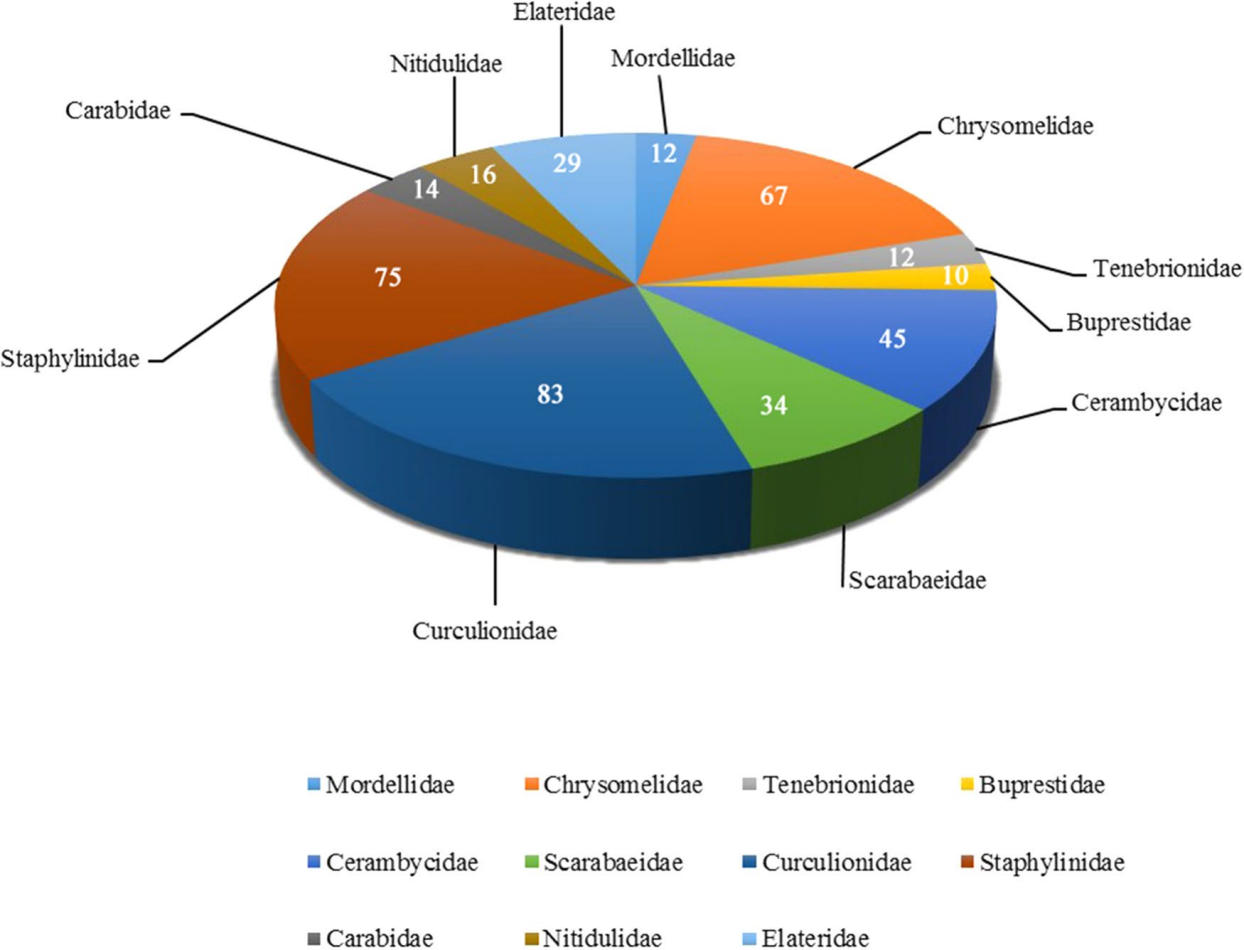
Families with more than ten species (a total of 11 families) were selected for presentation. The sample sizes of each groups were also shown.

Finally, data show the beetles’ biodiversity collected from each sampling site. Firstly, we summarized the data from each sampling point after completing the data statistics. Afterward, we counted the number of beetle individuals collected under the different passive acquisition methods at different points (Fig. 4). In Fig. 4, red, blue, and green represent the number of beetle individuals collected by MT, PT, and FIT, respectively. Fig. 4 shows that MT collected fewer beetles than FIT and PT. Secondly, the data of 13 sampling points in each collecting method were summarized to obtain the total number of families and species collected by each method (Fig. 5). A graph created in Excel 2016 displays the collection method as the horizontal coordinate and the number as the vertical coordinate. In the graph, red represents the number of families, and blue represents the number of species. Fig. 5 shows that FIT collected more beetle species and individuals than PT and MT, and MT collected the least. Thirdly, all data from the 13 sampling points and the three collection methods were summarized. The number of species collected in all families was counted. Families with more than ten species were selected (a total of 11 families) for data presentation (Fig. 6). Finally, a graphic was drawn in Excel 2016. Fig. 6 shows that the number of species in Staphylinidae, Curculionidae, and Chrysomelidae accounted for a large number, and the diversity was relatively high.

## Data Records

A master file containing photos and collected statistics are available on Dryad^22^ (10.5061/dryad.sn02v6x5c). It consists of the following parts:

1. One file (.docx) contains a summary of information on latitude, longitude, elevation, temperature, and rainfall for each month of 2019 in the Tei Tong Tsai area, as well as information on vegetation cover and degree of human disturbance at the 13 sample sites. After compression, the file is named Supplement 1. zip.
2. A morphological species identification chart is named Supplement 2. zip.
3. A folder containing 318 original photos (.JPG). After compression, the file name is Supplement 3. zip. Three folders are named after the collection methods. For example, in the DTZ-FIT file, DTZ is the acronym for the Chinese name of the sampling site, and FIT is the collecting method. Each folder was divided into different subfolders according to the collection date. Each subfolder is named with encoded information, for example, 2019DTZ-FIT-504–507, where 2019 represents the year for collection, DTZ is the acronym for the Chinese name of Tei Tong Tsai, FIT is the collecting method, and 504–507 represents the exact collection period from 4^th^ May to 7^th^ May. The photos under each folder are named, for example, 2019DTZ-FIT1-504-507, where 1 represents the number of sampling points. The other parts are described the same as above.
4. Three statistical data tables (.xlsx) of the number of beetles with the three passive acquisition methods. After compression, the file name is Supplement 4. zip. The three tables (.xlsx) include the original statistical dataset of the beetles collected from the Tei Tong Tsai. Each table has 13 sheets, and each sheet was named following the sampling point order. The information in these tables shows the family and species names of beetles in the first and second columns. For example, a headline is named Tei Tong Tsai-(2019)-FIT-1, where Tei Tong Tsai represents the collecting sample site, 2019 represents the collection year, FIT is the collection method, and 1 represents the number of sampling points. The second line shows detailed collection information on the sampling points; for example, 2019DTZ-FIT1-510-512, where 2019 represents the collection year, DTZ is the acronym for the Chinese name of the sample site, FIT is the collection method, 1 represents the collection of sampling points, and 510–512 represents the exact collection period from 10^th^ May to 12^th^ May.

## Technical Validation

All specimens were photographed in a standardized environment with an Olympus EM5 (60 mm) camera. Data statistics were performed by the same researcher referring to the same reference material to ensure minimal human interference in the experiment. All sample statistic sets were checked twice for any possible errors.

## Usage Notes

This manuscript can serve as a reference for species diversity research on beetles in Hong Kong. The aim of this dataset is to provide data support for studying beetle species diversity in Hong Kong. This dataset can also obtain differences between the three collection methods by comparing the numbers of collected beetle species and individuals. In addition, the three passive acquisition methods occupy different vertical spaces, emphasizing the usefulness of this dataset for evaluating differences in the morphology, species, and numbers of beetles in different habitats. Furthermore, the external morphology supports quantitative analysis such as geometric morphometrics and mathematical morphology.

## Data Availability

No code was used in this study.
